# Artificial intelligence-detected HER2 strong-positive tumor proportion predicts FISH positivity and treatment response in breast cancer

**DOI:** 10.1371/journal.pone.0352979

**Published:** 2026-07-06

**Authors:** Seung Geun Song, Soo Ick Cho, Taeyong Sim, Sangwon Shin, Wonkyung Jung, Jisoo Shin, Sukjun Kim, Heon Song, Sérgio Pereira, Donggeun Yoo, Sun Kyung Baek, Kiyong Na, Minsun Jung, So-Woon Kim

**Affiliations:** 1 Department of Pathology, Seoul National University College of Medicine, Seoul, Republic of Korea; 2 Lunit, Seoul, Republic of Korea; 3 Department of Internal Medicine, Kyung Hee University College of Medicine, Seoul, Republic of Korea; 4 Department of Pathology, Kyung Hee University Hospital, Kyung Hee University College of Medicine, Seoul, Republic of Korea; 5 Department of Pathology, Yonsei University College of Medicine, Seoul, Republic of Korea; Konkuk University, KOREA, REPUBLIC OF

## Abstract

Human epidermal growth factor receptor 2 (HER2)-targeted therapies have revolutionized breast cancer treatment, necessitating standardized HER2 testing. However, current immunochemistry-based HER2 assessment faces challenges due to subjectivity and variability among observers, prompting the guidance of artificial intelligence (AI). We evaluated AI’s efficacy in HER2 status assessment and treatment response prediction, especially focusing on complete and intense circumferential HER2-positive (3+) tumor cells. An AI-powered HER2 analyzer (Lunit SCOPE HER2, Lunit Inc., Seoul, South Korea) and three pathologists independently assessed HER2 3 + tumor cell proportions from whole-slide images of 191 breast cancer cases from Kyung Hee University Hospital. Logistic regression and receiver operating characteristic (ROC) curves determined predictive accuracy. AI-detected 3 + tumor cell proportions strongly correlated with fluorescent in situ hybridization (FISH) positivity (area under ROC curve [AUC]: 0.783) and pathological complete response (pCR) rates (odds ratio [OR]: 1.003–1.052), outperforming pathologists’ assessments. Combining AI improved prediction accuracy of pathologists, with an AUC increased from 0.712–0.813 to 0.790–0.821 for predicting FISH positivity and median OR increased from 0.996–1.061 to 1.003–1.055 for predicting pCR. Overall, this preliminary study suggests that AI could enhance HER2 status determination and treatment response prediction, complementing traditional pathological evaluation of HER2 immunohistochemistry.

## Introduction

Human epidermal growth factor receptor 2 (HER2)-targeted treatments have significantly transformed the breast cancer treatment landscape, leading to the standardization of HER2 testing as a crucial procedure for all patients, which now plays a vital role in guiding treatment decisions and providing predictive insights [1].

The American Society of Clinical Oncology (ASCO)/College of American Pathologists (CAP) guidelines categorize HER2 status by examining immunohistochemistry (IHC) staining intensity and the proportion of positive cells. In cases indicating equivocal HER2 status, further validation through in situ hybridization (ISH) testing is essential to ascertain the presence of *HER2* gene amplification [1].

While IHC-based HER2 testing serves as a cornerstone in current HER2-targeted therapies, the inherent subjectivity and variability of HER2 testing have led to concerns over its reliability, sparking a considerable interest in leveraging artificial intelligence (AI)-powered tools for a more accurate assessment of HER2 status [2,3]. Furthermore, recent results from the DESTINY-Breast04 and DESTINY-Breast06 studies have expanded the eligibility for HER2-targeted treatment to include patients with HER2 low-expressing tumors, such as 1+ and 2 + , highlighting the increasing importance of elaborate quantitative assessment of HER2 beyond the current ASCO/CAP guideline [4,5].

In this preliminary study, we explored how AI-based quantitative scoring of HER2 intensity could assist in assessing FISH positivity and response to HER2-targeted therapy. Interestingly, we found that AI-determined 3 + HER2 scoring was particularly significant in predicting fluorescence ISH (FISH) positivity and in assessing the response to HER2-targeted therapy.

## Materials and methods

### AI tool and output definition

In this study, we applied an AI-powered HER2 analyzer for breast cancer, Lunit SCOPE HER2 (Lunit Inc., Seoul, Republic of Korea), to assess the proportion of 3 + tumor cells from a whole-slide image (WSI) of breast cancer as previously reported [6]. The AI analyzer classifies tumor-cell membranous HER2 immunoreactivity according to the ASCO/CAP framework as follows: 3+ (complete and intense circumferential membrane staining), 2+ (weak to moderate complete membrane staining), 1+ (incomplete membrane staining that is faint/barely perceptible), and 0 (no staining) [1]. In this study, the primary output was the proportion (%) of tumor cells classified as 3 + . Briefly, the software integrates a cell-level module that identifies tumor cells and assigns HER2 intensity classes (0/1 + /2 + /3+) with a tissue-level module that segments carcinoma regions, enabling slide-level quantification of 3 + tumor cells. Detailed model architecture and training procedures are provided in Supporting Information (S1 Text: Supplementary Methods).

### Study cohort and whole-slide image preparation

This retrospective study was conducted using medical records obtained from Kyung Hee University Hospital (Seoul, Republic of Korea). The dataset was accessed on 4 February 2023 for research purposes. During data collection and analysis, the investigators did not have access to any personally identifiable information. The external test set was selected from Kyung Hee University Hospital. Inclusion criteria were pathology-proven breast cancer diagnosed between September 2004 and August 2022, availability of matched HER2 IHC whole-slide images, and either available FISH testing or receipt of neoadjuvant HER2-targeted therapy (see Supplementary Table 1 in S1 File for case characteristics). All slides were digitized using either a P1000 scanner (3DHistech, Budapest, Hungary) or an Aperio AT2 (Leica Biosystems, Wetzlar, Germany).

Whole-slide image selection and clinical endpoints (FISH results and pCR outcomes) were obtained from routine clinical care at the hospital and were not generated by the commercial sponsor.

### Pathologist reader study

Three board-certified pathologists independently scored the proportion of 3 + tumor cells in the same WSIs, without AI assistance. Pathologists assessed the proportion of 3 + tumor cells using a digital pathology viewer and recorded estimates using predefined increments (0.5% for ≤5%, 1% for >5% to ≤15%, and 5% for >15%) to standardize reporting. For AI-assisted reading analyses, the AI output was combined with each pathologist’s estimate using the harmonic mean, and performance was re-evaluated using the same endpoints.

### Endpoints and statistical analysis

The proportion of 3 + tumor cells was used to predict FISH positivity and outcomes after neoadjuvant HER2-targeted therapy (Miller and Payne system: pathological complete response [pCR] versus others) [7]. Receiver operating characteristic (ROC) curves were used to compare AI and pathologist estimates of 3 + tumor proportions for predicting FISH results. AUCs and confidence intervals (CIs) were estimated using the DeLong method. For a clinically interpretable threshold analysis, we evaluated prediction performance using the > 10% 3 + cut-off (clinical HER2 IHC 3 + definition) and reported overall accuracy, PPV, and NPV; an ROC-derived optimal threshold was additionally used for exploratory analyses. The optimal threshold was defined as the point that maximized sensitivity and specificity on the ROC curves. We used logistic regression to determine the significance and odds ratios (ORs) of 3 + tumor proportions in predicting pCR status. Non-parametric comparisons between groups were performed using the Mann-Whitney U test where applicable. All analyses were conducted in Python (version 3.10.12). De-identified, case-level data underlying the main analyses (including FISH status, clinical IHC score, AI-derived 3 + measurements, and pathologist-estimated 3 + proportions) are provided as Supporting Information (S1 Data).

### Ethics statement

This study was approved by the Institutional Review Board of Kyung Hee University Hospital (no. KHUH 2022-01-035). As this retrospective study used only materials collected for patient diagnosis and posed no additional risk to patient safety or privacy, the requirement for informed consent was waived.

## Results

### HER2 expression level and clinical outcomes

A total of 191 breast cancer cases and matched HER2 WSIs were included, with a median age at diagnosis of 58 years and an initial stage of II being the most common (n = 72, 37.7%). Detailed demographic and clinical information are provided in Supplementary Table 1 in S1 File. Among 170 cases with FISH results, 35 (20.6%) were FISH positive. There were 25 cases of HER2-targeted neoadjuvant therapy (12 [48.0%] of the TCHP regimen and 13 [52.0%] of the AC-TH regimen), of which 10 (40.0%) were pCR.

### Prediction of FISH positivity using the proportion of HER2 3 + tumor cells

**Fig 1:**
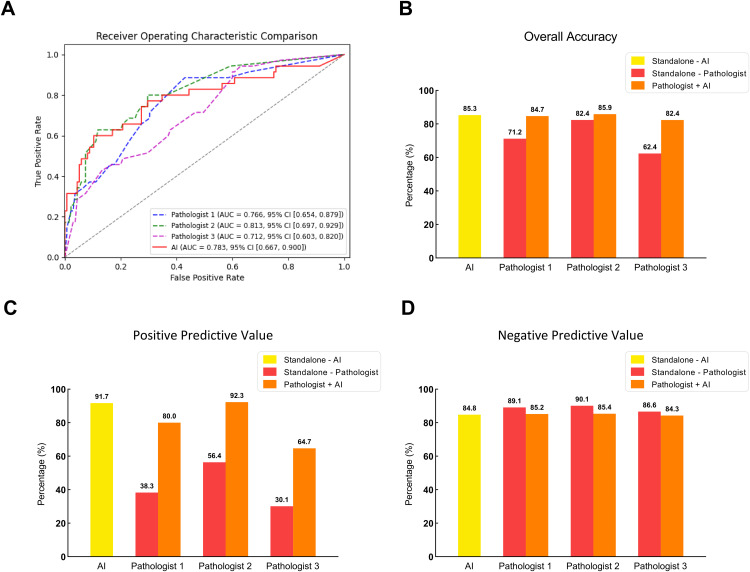
Performance comparison of pathologists and AI in HER2 staining quantification and FISH positivity prediction. **A** ROC curves showing the estimated proportion of HER2 3 + intensity tumor cells and corresponding FISH positivity, comparing standalone performance between pathologists and AI. **B-D** FISH positivity prediction based on a 10% cutoff for HER2 3 + tumor cells (HER2 IHC 3 + score per ASCO/CAP guidelines). Each pathologist’s standalone and combined performance with AI (calculated by harmonic mean) is presented in the following metrics: **B** Overall accuracy, **C** Positive predictive value, and **D** Negative predictive value. AI, artificial intelligence; AUC, area under curve; CI, confidence interval.

The mean proportion of 3 + tumor cells identified by AI was significantly higher in the FISH-positive group (19.2 ± 33.0%) compared to the FISH-negative group (0.8 ± 2.5%) (p < 0.001). This trend was similarly observed in assessments by three pathologists (p < 0.001) (Supplementary Table 2 in S1 File). For predicting FISH positivity, the proportion of 3 + tumor cells detected by AI yielded an area under the ROC curve (AUC) of 0.783 (95% CI: 0.667–0.900) (Fig 1A). The optimal threshold for FISH prediction was determined to be 1.331% for the 3 + tumor cell ratio, with a sensitivity of 0.600 and specificity of 0.896. The pathologists’ evaluations showed AUCs of 0.766 (95% CI: 0.654–0.879), 0.813 (95% CI: 0.697–0.929), and 0.712 (95% CI: 0.603–0.820), respectively. When divided by scanner, AI showed similar predictive power in AUC values for both scanners (Supplementary Fig 1A and 1B in S1 File).

When FISH positivity was predicted using a 10% threshold for the proportion of 3 + tumor cells (aligning with HER2 IHC 3+ by ASCO/CAP guidelines) [1], the AI model achieved an overall accuracy of 85.3%, with a positive predictive value (PPV) of 91.7% and a negative predictive value (NPV) of 84.8% (Fig 1B-1D). In contrast, the pathologists showed lower PPVs and higher NPVs, resulting in overall accuracies lower than that of the AI model (Fig 1B-1D). Analysis of the original clinical HER2 IHC reports showed that 1 of 9 cases (11.1%) classified as HER2 IHC 3+ (>10% of tumor cells with complete and intense circumferential membrane staining) was FISH-negative. A full 2x2 contingency table for the > 10% cutoff analysis (3 + proportion >10% vs. FISH) is provided in Supplementary Table 3 in S1 File, and the underlying case-level measurements are provided in S1 Data.

When predicting FISH positivity by combining the 3 + tumor cell proportions determined by each pathologist and the AI model using the harmonic mean, AUCs improved for all pathologists (from 0.766 [95% CI: 0.654–0.879] to 0.796 [95% CI: 0.681–0.911], from 0.813 [95% CI: 0.697–0.929] to 0.821 [95% CI: 0.704–0.939], and from 0.712 [95% CI: 0.603–0.820] to 0.790 [95% CI: 0.674–0.906]) (Supplementary Fig 2 in S1 File). However, these differences were not statistically significant (p = 0.731, 0.781, and 0.450, respectively). Overall accuracy and PPV consistently improved for each pathologist when combined with the AI model (Fig 1B-1D).

### Prediction of neoadjuvant chemotherapy outcome by the proportion of HER2 3 + tumor cells

The mean proportion of 3 + tumor cells identified by the AI model was significantly higher in the pCR group (80.4 ± 31.8%) compared to the non-pCR group (38.0 ± 43.1%) (p = 0.023) (Supplementary Table 2 in S1 File). The pathologists’ evaluations also showed similar findings, although not statistically significant for one of them. In logistic regression analysis, the proportion of 3 + tumor cells identified by the AI model was significantly associated with pCR (OR=1.028, 95% CI: 1.003–1.052) (p = 0.026). In contrast, none of the evaluations by the three pathologists showed significant correlations with pCR. When predicting pCR using the harmonic mean of the pathologists and the AI model, statistically significant correlations with pCR were observed in all three pathologists (Table 1).

**Table 1:** Prediction of pathological complete response (pCR) status based on HER2 3 + proportion using logistic regression.

	Standalone		Pathologist + AI (Harmonic mean)	
	OR (95% CI)	p-value	OR (95% CI)	p-value
AI model	1.028 (1.003–1.052)	0.026	NA	NA
Pathologist 1	1.028 (0.996–1.061)	0.082	1.029 (1.003–1.055)	0.027
Pathologist 2	1.036 (0.998–1.076)	0.065	1.029 (1.003–1.055)	0.028
Pathologist 3	1.101 (0.957–1.267)	0.177	1.029 (1.003–1.055)	0.028

OR, odds ratio; CI, confidence interval.

## Discussion

This study explored the use of AI in assessing HER2 status and predicting treatment response in breast cancer patients, specifically focusing on the percentage of 3 + tumor cells. AI’s evaluation of HER2 3 + tumor cell proportions showed stronger correlations with FISH positivity and pCR rates than pathologists’ assessments. Moreover, by combining the results of the AI to those of pathologists, the prediction results improved in all pathologists. These outcomes underscore AI’s potential in refining HER2 scoring and treatment outcome prediction.

The significant association we identified between the proportion of strong HER2-positive tumor cells and pCR suggests that quantitatively measuring HER2 protein expression using AI could provide a more detailed prediction of treatment response than the current semi-quantitative IHC scoring system alone, consistent with a previous report [8]. Interestingly, while individual pathologists’ assessments of the strong HER2-positive proportion showed no association with pCR in logistic regression models, a significant association emerged when their evaluations were averaged with that of the AI estimate using the harmonic mean. Although the odds ratios per 1%-point increase were numerically close to 1, they represent effects measured on a small unit scale. Accordingly, larger differences in the HER2 3 + tumor-cell proportion may correspond to more clinically meaningful differences in the odds of pCR; however, these findings should be interpreted cautiously given the small neoadjuvant cohort. These findings support the use of the AI system as a quantitative decision-support tool that provides standardized measurements of the HER2 3 + tumor-cell proportion and complements pathologists’ assessment within an integrated pathological and clinical evaluation.

Additionally, our results link AI-determined 3 + tumor cell proportion to FISH positivity, suggesting that AI-assisted detection could help pathologists accurately predict *HER2* amplification and might save some cases from unnecessary FISH testing. Although prior studies have established a correlation among HER2 IHC scores, FISH results, and treatment outcomes, these findings remain limited and inconclusive. For example, a retrospective analysis involving 560 cases of HER2-positive breast cancer reported a significantly higher pCR rate in patients with an IHC 3 + score compared to those with lower scores but *HER2* amplification by FISH (67% vs. 17%) [9]. Other researches showed unignorable discordance rates (up to 12%) between HER2 IHC 3+ and ISH positivity, which was particularly pronounced with IHC + /ISH- compared to IHC-/ISH+ cases [10], and FISH positivity in 14% of IHC 1 + cancers [11]. These findings suggest that the IHC scoring guideline may only partially reflect true *HER2* amplification status and potential treatment response. Moreover, the subjectivity of IHC results, variability among observers, and the limitations of ISH testing—including its availability, complexity, turnaround time, and cost—underscore the usability of AI as a reliable and accessible diagnostic method for HER2 testing [2].

We observed a small proportion of discordant cases between clinical HER2 IHC 3 + status (>10% strong complete membranous staining) and FISH results. This finding highlights that HER2 IHC scoring, while clinically informative, does not perfectly reflect gene amplification status in all cases. Therefore, confirmatory ISH testing remains important in selected scenarios, particularly when clinicopathologic findings are discordant or when treatment decisions require definitive amplification status.

Our study has some limitations. Given the disclosed competing interests, these findings should be interpreted with appropriate caution and further validated in independent, multi-center cohorts. Being retrospective and single-center, it inherently introduces potential confounders. Excluding 1+ and 2 + cells from evaluation deviates from the current guideline [1]; however, our analyses focused on the 3 + tumor-cell proportion because the primary objective was to assess its utility as a practical predictor of FISH positivity in routine settings. Additionally, the sample size, especially in the neoadjuvant group, was small. Because the evaluated AI tool is a commercial product, full disclosure of the underlying algorithm and training code is not available. Therefore, while our validation analyses are reproducible using the provided case-level dataset and pre-specified evaluation procedures, the model development process itself cannot be fully reproduced by external investigators.

In conclusion, our findings suggest that AI-based quantification of the HER2 3 + tumor-cell proportion may serve as a quantitative decision-support tool that complements pathologists’ assessment in estimating FISH positivity and treatment response. Further validation in larger independent cohorts is warranted before clinical implementation.

## Supporting information

S1 File Supplementary tables and figure. This file contains supplementary tables 1–3 and supplementary figs 1–2.
(PDF)

S1 Text Supplementary methods. Detailed model architecture and training procedures for the AI system.
(PDF)

S1 Data De-edentified case-level dataset underlying the main analyses. (CSV)
