# Supplementary material for: Artificial intelligence-detected HER2 strong-positive tumor proportion predicts FISH positivity and treatment response in breast cancer
Source: PLoS One. 2026 Jul 6;21(7):e0352979. doi: 10.1371/journal.pone.0352979 (PMC13336205; doi:10.1371/journal.pone.0352979)

Supplementary Information

Supplementary Tables

Supplementary Table 1. Demographics and clinical information of the patient group

	All cases (n = 191)	Having FISH results (n = 170)	HER2-targeted neoadjuvant therapy (n = 25)
Sex<br>Female	191 (100%)	170 (100%)	25 (100%)
Age (median [min, max])	58.0 (28.0, 92.0)	58.0 (28.0, 92.0)	53.0 (43.0, 68.0%)
HER2 status (EMR)<br>3+<br>2+<br>1+	28 (14.7%)<br>162 (84.8%)<br>1 (0.5%)	9 (5.3%)<br>160 (94.1%)<br>1 (0.6%)	19 (76.0%)<br>6 (24.0%)<br>0 (0.0%)
Initial stage<br>I (A/B)<br>II (A/B)<br>III (A/B/C)<br>IV	 70 (36.6%)<br>72 (37.7%)<br>32 (16.8%)<br>17 (8.9%)	 65 (38.2%)<br>66 (38.8%)<br>23 (13.5%)<br>16 (9.4%)	 11 (44.0%)<br>11 (44.0%)<br>1 (4.0%)<br>2 (8.0%)
Scanner<br>P1000<br>AT2	 131 (68.6%)<br>60 (31.4%)	 128 (75.3%)<br>42 (24.7%)	 6 (24.0%)<br>19 (76.0%)

EMR, electronic medical record

Supplementary Table 2. The mean proportion of HER2 3+ tumor cells by the artificial intelligence (AI) model and pathologists according to the fluorescence in situ hybridization (FISH) result or Miller-Payne criteria after neoadjuvant therapy

	FISH result			Pathological complete response (pCR)		
	FISH positive (n = 35), %±SD	FISH negative (n=135), %±SD	p-value	Yes (n =10), %±SD	No (n=15), %±SD	p-value
AI model	19.2±33.0	0.8±2.5	<0.001	80.4±31.8	38.0±43.1	0.023
Pathologist 1	38.5±37.4	11.7±20.8	<0.001	91.5±25.2	62.8±40.0	0.090
Pathologist 2	35.5±37.2	6.5±16.5	<0.001	95.0±15.8	60.7±43.1	0.024
Pathologist 3	44.5±39.5	19.9±27.3	<0.001	99.0±3.16	66.8±38.0	0.020

SD: standard deviation

Supplementary Table 3. 2x2 contingency table for predicting FISH positivity using the >10% cut-off for the proportion of HER2 3+ tumor cells (standalone AI, standalone pathologists, and AI-assisted pathologists).

Method	TP	FP	FN	TN	N	Overall accuracy (%)	PPV (%)	NPV (%)
AI (>10% 3+)	11	1	24	134	170	85.3	91.7	84.8
Pathologist 1 (>10% 3+)	23	37	12	98	170	71.2	38.3	89.1
Pathologist 2 (>10% 3+)	22	17	13	118	170	82.4	56.4	90.1
Pathologist 3 (>10% 3+)	22	51	13	84	170	62.4	30.1	86.6
Pathologist 1 + AI (harmonic mean, >10%)	12	3	23	132	170	84.7	80	85.2
Pathologist 2 + AI (harmonic mean, >10%)	12	1	23	134	170	85.9	92.3	85.4
Pathologist 3 + AI (harmonic mean, >10%)	11	6	24	129	170	82.4	64.7	84.3

FISH positivity was used as the reference standard. The >10% cut-off for HER2 3+ tumor-cell proportion corresponds to the ASCO/CAP HER2 IHC 3+ definition. AI-assisted values were calculated using the harmonic mean of each pathologist's estimate and the AI estimate. One case with FISH testing performed but missing result was excluded (N=170). Abbreviations: TP, true positive; FP, false positive; FN, false negative; TN, true negative; PPV, positive predictive value; NPV, negative predictive value; FISH, fluorescence in situ hybridization; AI, artificial intelligence.

Supplementary Figures

Supplementary Fig 1: ROC curves for predicting FISH positivity based on the proportion of HER2 3+ intensity tumor cells, categorized by scanner type.

A ROC curve for P1000 (n=128): AUC 0.772 (95% CI: 0.627–0.917). **B** ROC curve for Aperio AT2 (n=42): AUC 0.773 (95% CI: 0.574–0.972). AI, artificial intelligence; AUC, area under curve; CI, confidence interval.

A

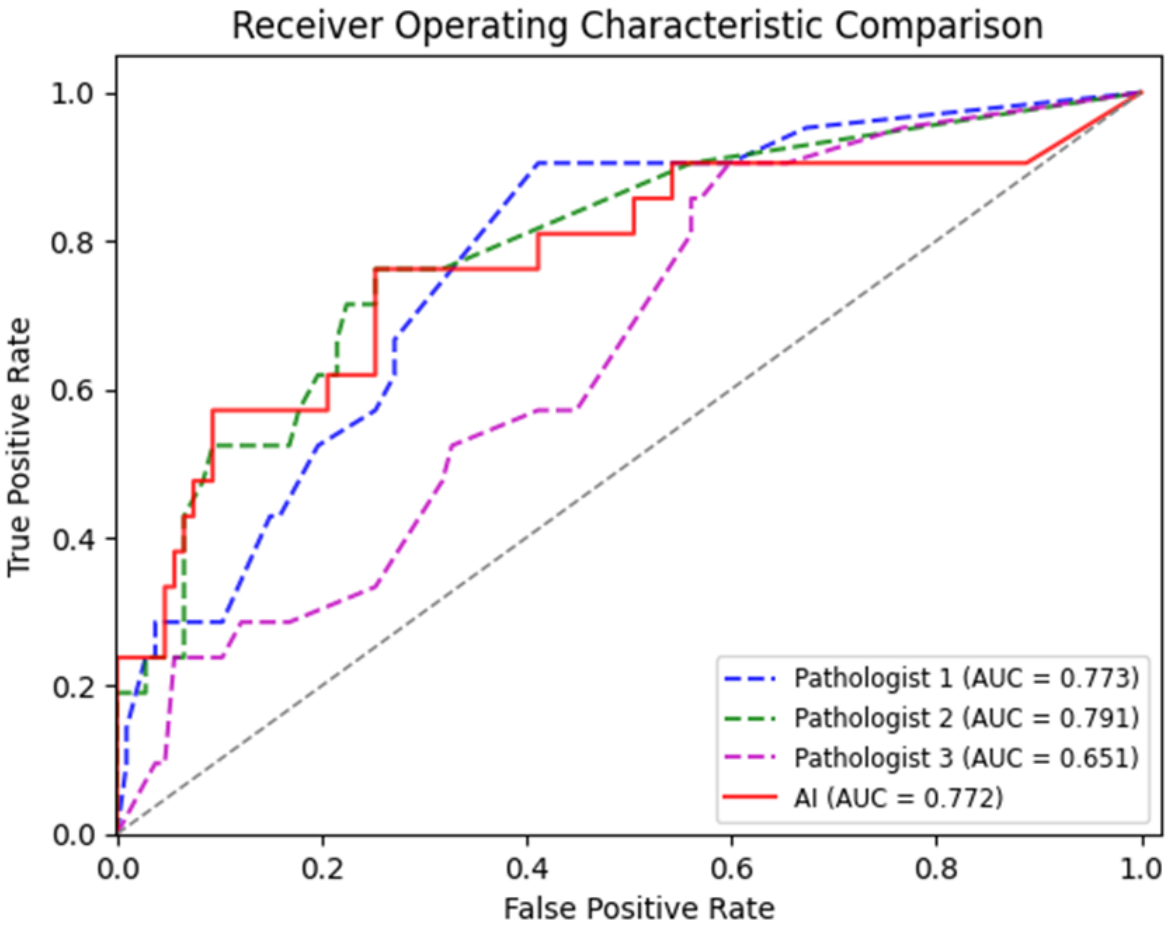

B

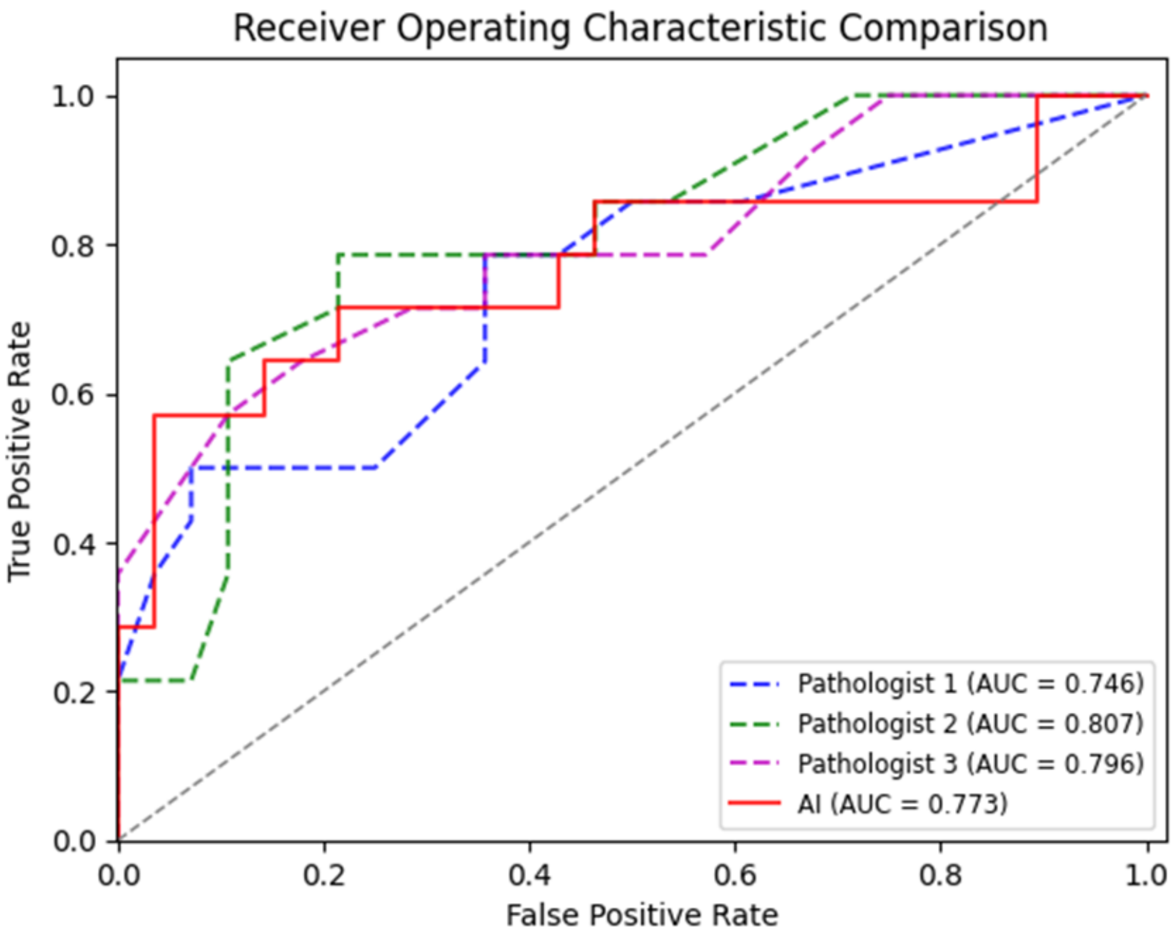

Receiver Operating Characteristic Comparison
True Positive Rate
False Positive Rate
0.0
0.2
0.4
0.6
0.8
1.0
Pathologist 1 (AUC = 0.746)
Pathologist 2 (AUC = 0.807)
Pathologist 3 (AUC = 0.796)
AI (AUC = 0.773)

Supplementary Fig 2: ROC curves for FISH positivity estimation by AI-assisted quantification of HER2 3+ intensity tumor cell proportion for each pathologist. Assessment for each case was combined with AI results via the harmonic mean. AI, artificial intelligence; AUC, area under curve; CI, confidence interval.

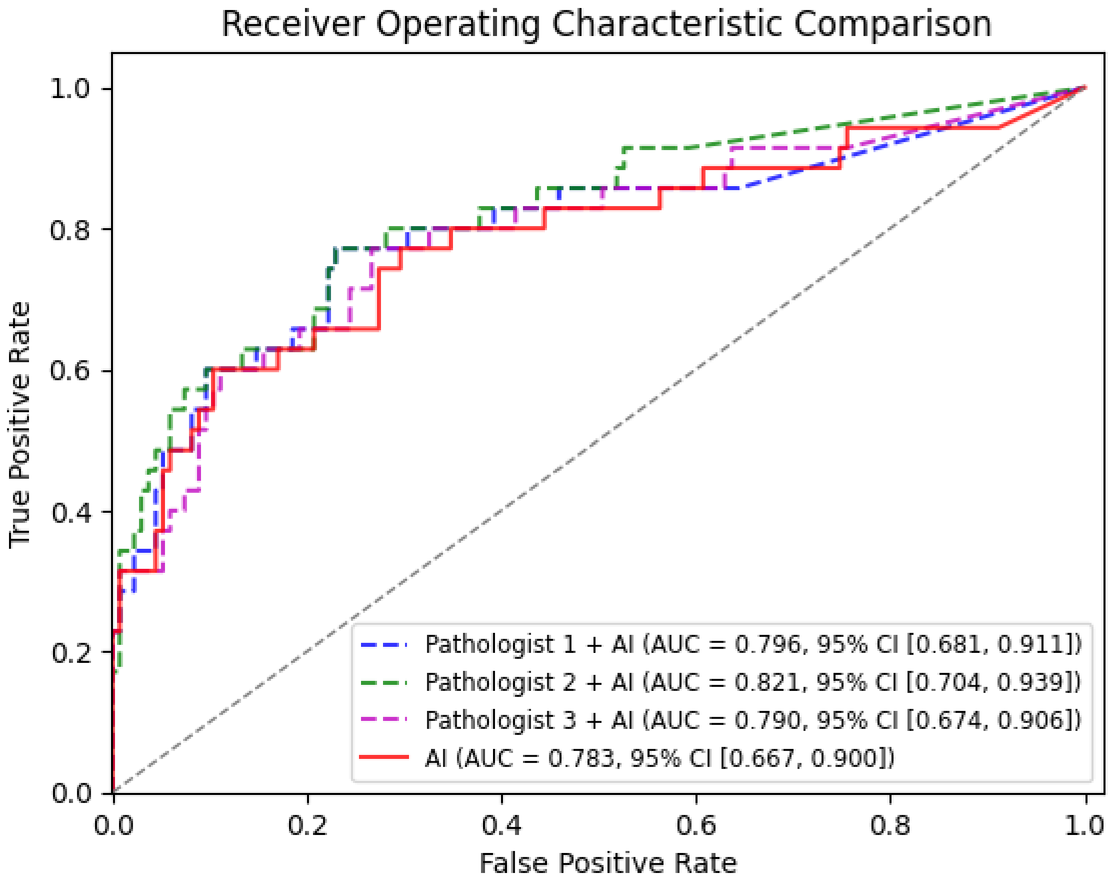

Supplement: S1 File — This file contains supplementary tables 1–3 and supplementary figs 1–2. (PDF) [file pone.0352979.s001.pdf]
