# Supplementary material for: Artificial intelligence-detected HER2 strong-positive tumor proportion predicts FISH positivity and treatment response in breast cancer
Source: PLoS One. 2026 Jul 6;21(7):e0352979. doi: 10.1371/journal.pone.0352979 (PMC13336205; doi:10.1371/journal.pone.0352979)
Supplement: S1 Text — Detailed model architecture and training procedures for the AI system. (PDF) [file pone.0352979.s002.pdf]

Supplementary Methods

AI model architecture and training

The HER2 analysis platform, Lunit SCOPE HER2 (Lunit, Seoul, Republic of Korea), was constructed using a dataset of 1,259 breast cancer whole-slide images (WSIs) stained for HER2 by immunohistochemistry, partitioned into training (n=880), tuning (n=253), and internal validation (n=126) subsets. From these WSIs, image patches of predetermined physical areas (0.04 mm² for cellular regions and 2.54 mm² for tissue regions) were generated and normalized to a resolution of 0.19 µm per pixel, resulting in 1024×1024–pixel cell patches and 8192×8192–pixel tissue patches. To avoid cross-contamination among datasets, patch inclusion strictly adhered to the WSI-level split. A total of 8,149 cell patches and 4,443 tissue patches were used for model development, encompassing annotations for 813,132 tumor cells, 804,914 non-tumor cells, 101,183.67 mm² of invasive carcinoma, and 2,653.89 mm² of carcinoma in situ. All extracted patches underwent expert annotation by board-certified pathologists.

At the cellular scale, the model discriminated between tumor and non-tumor cells and assigned tumor cells to one of four immunoreactivity levels (3+, 2+, 1+, 0). This segmentation-driven classification was implemented using a DeepLabv3+ architecture supported by a ResNet-34 encoder. The accompanying tissue-level model performed pixel-wise assignment to invasive carcinoma (CA), carcinoma in situ (CIS), or background (BG), leveraging a DeepLabv3 network with a ResNet-101 backbone. Slide-wide HER2 assessment was derived by integrating results from the cell and tissue modules, allowing quantification of HER2-positive tumor cells within CA regions and estimation of the proportional distribution of the four HER2 intensity

classes. The final categorization of HER2 expression for each slide followed the interpretive framework recommended by the American Society of Clinical Oncology (ASCO) and the College of American Pathologists (CAP).
